# A Novel Cobalt-Activated Halotolerant α-Amylase with High Specific Activity from *Priestia* sp. W243 in Kuwait Sabkha for Biotechnological Applications

**DOI:** 10.3390/md24020065

**Published:** 2026-02-03

**Authors:** Surendraraj Alagarsamy, Sabeena Farvin Koduvayur Habeebullah, Ismail Saheb Azad, Saja Adel Fakhraldeen, Turki Al Said, Aws Al Ghuniam, Faiza Al-Yamani

**Affiliations:** Coastal and Marine Resources Program, Environment and Life Sciences Research Center, Kuwait Institute for Scientific Research, P.O. Box 1638, Salmiya 22017, Kuwait; shabeebullah@kisr.edu.kw (S.F.K.H.); aismail@kisr.edu.kw (I.S.A.); sfakhraldeen@kisr.edu.kw (S.A.F.); trsaid@kisr.edu.kw (T.A.S.); aghunaim@kisr.edu.kw (A.A.G.); faizayamani@gmail.com (F.A.-Y.)

**Keywords:** sabhkas, halophilic bacteria, halotolerant α-amylase, enzyme purification, cobalt-dependent metalloenzyme

## Abstract

Sabkhas, hypersaline ecosystems along Kuwait’s coastal zone, are extreme environments that harbor diverse halophilic microorganisms with significant biotechnological potential. Despite this, they remain underexplored, particularly in the context of enzymes that can function under high salinity. The aim of this study is to identify bacterial isolates from Kuwait’s sabkhas that produce α-amylase under extreme environmental conditions and to purify and characterize the resulting halotolerant α-amylase. Among the seven α-amylase-producing isolates, *Priestia* sp. W243, isolated from Mina Abdullah, exhibited the highest enzyme production under optimal growth conditions of pH 9.0, 37 °C, and 7.5% NaCl. A novel halotolerant α-amylase with a remarkably high specific activity (8112.1 U/mg) was purified from this isolate using ultrafiltration, ion-exchange chromatography, and gel-filtration. The purified enzyme, with a molecular weight of 25 kDa, showed optimal activity at 40 °C, pH 8, and 3% NaCl. Notably, the enzyme remained active in the absence of salt and up to 15% NaCl, demonstrating exceptional halotolerance. Metal ion profiling revealed that enzyme activity was significantly enhanced by Co^2+^, whereas Ca^2+^ had a comparatively moderate effect on enzyme activity. When the effects of metal chelators were examined, EDTA, a strong metal chelator, inhibited the enzyme. However, the enzyme remained active when Ca^2+^ was specifically removed using EGTA, suggesting that this α-amylase may be a cobalt-dependent metalloenzyme, which is an unusual characteristic among known α-amylases. Additionally, the enzyme retained its catalytic activity under reducing conditions (e.g., in the presence of DTT and β-mercaptoethanol), indicating structural stability is independent of disulfide bonds. These unique properties distinguish this α-amylase from typical salt- or calcium-dependent counterparts and highlight its potential for industrial applications in high-salt food processing, baking, brewing, and environmental remediation.

## 1. Introduction

The global enzymes market, valued at $12.5 billion in 2022, is experiencing rapid growth, with a projected compound annual growth rate of 6.1% [1]. Microbial enzymes account for over 75% of market demand and are at the forefront of this expansion. Among these, α-Amylase (1,4-alpha-D-glucan-glucanohydrolase, EC 3.2.1.1), which hydrolyzes starch by breaking α-1,4-glucosidic bonds, is an important industrial enzyme. It accounts for approximately 30% of the global enzyme market due to its high demand in the starch industry and various other industries, such as food, beverages, pharmaceuticals, textiles, and detergents [2]. Given the varying reaction conditions required for starch hydrolysis in different industrial applications, enzymes with exceptional catalytic activity and stability under extreme conditions such as high or low temperatures, extreme pH levels, and high salinity are highly valued. Such enzymes are ideal for processes that demand specific and often challenging environmental parameters [3,4]. Consequently, numerous thermophilic, psychrophilic, acidophilic, and alkaliphilic α-amylases have been isolated from microorganisms in extreme environments, offering diverse industrial applications [5]. By 2030, enzymatic catalysis is expected to replace up to 40% of chemical synthesis processes that rely on environmentally harmful solvents and high-energy inputs, significantly boosting the demand for novel industrial enzymes [6]. In this context, the exploration and development of extremozymes are crucial to meet the growing industrial demand for sustainable and efficient catalytic processes.

Sabkhas, typical hypersaline environments, are prominent surface features in Kuwait’s coastal zone, covering approximately 769.4 km^2^, equivalent to 4.3% of the nation’s total area [7]. These unique ecosystems are found in Al-Julai’ah, Al-Khiran, Al-Maqwa, Urafjan, and Al-Gurain areas. These hypersaline environments are characterized by extreme conditions such as high salinity, exposure to high and low temperatures, low oxygen conditions, and, in some cases, high pH values [7,8]. Despite these harsh conditions, sabkhas harbor diverse microbial communities, with halophilic bacteria and archaea being the most prevalent organisms [8,9]. Halophilic microorganisms, often poly-extremophiles, have been reported to possess significant biotechnological potential and produce a range of valuable substances, including pigments, hydrocarbon-degrading agents, polyhydroxyalkanoates, halocins, exopolysaccharides, bioemulsifiers, and novel extremozymes [3,4]. Their ability to thrive in extreme conditions makes these microorganisms ideal candidates for cost-effective, contamination-resistant, fermentation-based biomolecule production [10]. These microorganisms’ unique biochemical and metabolic adaptations enable them to produce enzymes with enhanced solubility and functionality under low-water activity conditions [11]. Despite this potential, studies from the sabkhas of Kuwait and Saudi Arabia have been mostly restricted to microbial diversity and hydrocarbon degradation [8,9,12,13,14]. Given their extreme environmental conditions, Kuwait sabkhas could be a potential hotspot for novel extremozymes with industrial applications, which have remained unexplored. In our earlier study, 41.3% of halophiles isolated from these sabkhas exhibited amylase activity [9]. The optimum conditions for amylase production from the selected isolates were determined in this study. Accordingly, this work aims to identify a halotolerant bacterium with high α-amylase activity from Kuwait’s sabkha and to purify and characterize a novel halotolerant α-amylase with high specific activity and stability under extreme salinity conditions for potential industrial applications.

## 2. Results and Discussion

### 2.1. Identification of Bacterial Isolates with High Amylase Activity

The seven most potent isolates with amylase activity from sabkha stations in Bnaider, Mina Abdulla, Al-Zour, and Bubiyan Island were identified through 16S rRNA gene sequencing. BLAST (BLAST+, version 2.13.0) analysis revealed that all the amylase-producing isolates from Kuwait’s sabkhas exhibited 82.93% to 91.97% similarity with previously identified bacterial species from other parts of the world (Table 1). Considering the low levels of similarity, re-sequencing with high-quality reads is required for accurate taxonomic resolution or to identify them as a novel species. Accordingly, these isolates were identified only up to the genus level as *Bacillus* sp. W225, *Bacillus* sp. W365, *Vibrio* sp. W10, *Pseudomonas* sp. W226, *Halomonas* sp. W293, *Priestia* sp. W243, and *Marinobacter* sp. B28 (Table 1). Most were isolated from sediment samples, except for *Halomonas* sp. W293, obtained from seawater at Mina Abdulla. Although most isolates grew on marine agar with 3% salt, *Marinobacter* sp. (B28), a halophile, thrived at 15% salt concentration.

These findings are consistent with prior reports from various environments [3,4,15]. *Bacillus* spp. were known for robust enzyme production in industrial applications, while *Pseudomonas* species thrive in diverse environments, producing a range of biocatalysts [4,15,16]. *Halomonas* spp. were reported to produce salt-tolerant enzymes, which are valuable in biotechnology [4]. Earlier studies from Kuwait’s hypersaline environments recovered *Marinobacter* spp. and were reported to have hydrocarbon-degrading capabilities [13]. *Priestia flexa* isolated from a mangrove ecosystem was reported to accumulate high quantities of poly(3-hydroxybutyrate) (PHB), illustrating the diverse metabolic capabilities of bacteria from extreme environments [17]. These studies underscore the potential for discovering novel metabolic pathways and products from isolates obtained from the Kuwait sabkha environments.

### 2.2. Growth and α-Amylase Production of the Seven Selected Isolates at Different Temperatures, pH and Salt Concentrations

**Figure 1:**
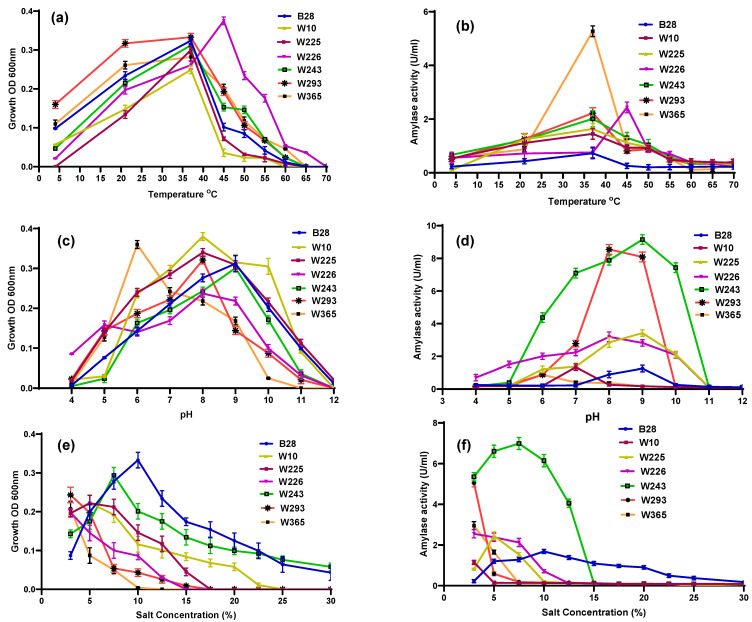
Growth and amylase activity of selected isolates from Kuwait Sabkha. Effect of temperature on growth (**a**) and amylase activity (**b**); effect of pH on growth (**c**) and amylase activity (**d**); and effect of salt on growth (**e**) and amylase activity (**f**) of the selected isolates.

The growth and enzyme production of the seven isolates under varying temperatures are shown in Figure 1a,b. As mesophiles, most isolates achieved peak growth at 37 °C with *Pseudomonas* sp. W226 displaying optimal growth at 45 °C. Figure 1b depicts amylase production across temperature ranges for these isolates, showing that maximum amylase levels aligned with their highest growth rates. Among the isolates, *Bacillus* sp. W365 had the highest amylase activity, followed by *Pseudomonas* sp. W226 and *Halomonas* sp. W293, with activities of 5.3, 2.4, and 2.2 U/mL, respectively. Studies have shown that the optimal temperature for amylase production in *Bacillus* spp. is generally around 37 °C [4,15,18]. Similar temperatures, typically between 37–40 °C, have also been reported for different *Pseudomonas* spp. [19,20] and *Halomonas* spp. [21,22], aligning with the results of this study.

Extracellular pH significantly influences α-amylase production by affecting bacterial cell membrane permeability, which can alter or inhibit the transport of cell constituents across the membrane [23]. The growth and enzyme production of the seven selected isolates at different pH levels are shown in Figure 1c,d. *Vibrio* sp. W10, *Halomonas* sp. W293, and *Pseudomonas* sp. W226 showed the highest growth at pH 8, while the two *Bacillus* spp., W365 and W225, demonstrated optimal growth at pH 6 and 8, respectively (Figure 1c). *Priestia* sp. W243 and *Marinobacter* sp. B28 exhibited the best growth at pH 9 (Figure 1c). In terms of amylase production, *Priestia* sp. W243, followed by *Halomonas* sp. W293 had the highest amylase production at pH 9 and 8 (9.1 and 8.5 U/mL), respectively (Figure 1d). *Priestia* sp. W243 showed strong amylase production across a broad pH range of 6–10, while *Halomonas* sp. W293 had high production within a pH range of 7–9 (Figure 1d).

Ubi et al. [24] reported a broader pH activity range (pH 4–11) for amylase production in *Priestia flexa* UCCM 00132. This adaptability to a wide pH range suggests that *Priestia flexa* can maintain enzymatic functionality across diverse environmental conditions, consistent with the high amylase production observed for *Priestia* sp. W243 in our study was observed at a pH range from 6 to 10. Although *Halomonas* spp. are known to grow across a wide pH range of 5 to 10, most studies report optimal pH for amylase production between 6.5 and 7 [25,26]. In contrast, the *Halomonas* sp. 293 isolate obtained from Kuwait sabkhas in our study showed maximum amylase production at pH 8. This higher optimal pH may be an adaptation to the unique saline and alkaline conditions of the sabkhas environment. The maximum amylase production observed for *Pseudomonas* sp. at pH 8 in our study supports the findings of Maalej et al. [27], although it slightly exceeds the optimal pH of 7.5 reported by Dutta et al. [19]. The optimal pH range for amylase production in various *Pseudomonas* species is generally reported to vary between 6.5 and 7, as seen in other studies [20,28]. The optimal pH range for amylase production in various *Bacillus* species has been reported to vary widely, from pH 5 to 11 [21,29], reflecting the adaptability of different *Bacillus* spp. to diverse environmental conditions.

Figure 1e,f show the growth and amylase production of the seven selected isolates under different salt concentrations. The highest growth was observed for *Marinobacter* sp. B28, followed by *Priestia* sp. W243, at 10 and 7.5% salt, respectively. In the case of *Vibrio* sp. W10 and *Bacillus* sp. W225, the growth was maximum at 5% salt, and for the other isolates, the growth was maximum at 3% salt. *Priestia* sp. W243 and *Marinobacter* sp. B28 showed a wide range of salt tolerance, exhibiting growth up to salt concentrations of 30%. *Bacillus* sp. W365 and *Pseudomonas* sp. W226 had a short tolerance range among the studied isolates (Figure 1e). Except for isolate W225, the amylase activity of all isolates showed direct correlation with their growth (Figure 1f). Isolate W243 (*Priestia* sp.), followed by W293 (*Halomonas* sp.), showed the highest amylase production at 7.5 and 3% salt concentration, respectively (Figure 1f). The *Vibrio* sp. isolate (W10) exhibited the lowest amylase production among all the isolates.

Though various studies have reported the isolation of amylase-producing *Priestia flexa* from marine environments such as mangrove soils and marine sediments [24,30,31], there are currently no specific studies on the optimal salt conditions for amylase production in *Priestia* sp., as it is an emerging genus in enzyme production research. Isolates *Priestia* sp. W243 and *Marinobacter* sp. B28 demonstrated amylase production across a broad salt concentration range (Figure 1f). Similar findings were reported for *Marinobacter* sp. isolated from Indian saline habitats, which exhibited growth and amylase production at salt concentrations as high as 20% (*w*/*v*), affirming the halophilic nature of these bacteria [31]. The highest amylase production was observed at a salt concentration of 3% for most isolates, including *Bacillus* sp. W225 and W365 (Figure 1f). *Bacillus* sp. has been reported to produce amylase across a broad range of salt concentrations, from 0% to 15% [18]. In the present study, *Halomonas* sp. W293 exhibited optimal amylase production at a salt concentration of 3%. A *Halomonas* sp. isolated from seaweed could thrive at salt concentrations up to 15%, with maximum amylase production observed at 8% [22]. While *Halomonas meridiana* from Spain showed optimal amylase production at 5% salt [25]. As moderate halophiles, *Halomonas* species are highly adaptable to varying salinity levels, making them valuable for industrial applications, particularly in enzyme production under saline conditions.

### 2.3. Selection of Best Isolate for Novel α-Amylase Isolation

**Figure 2:**
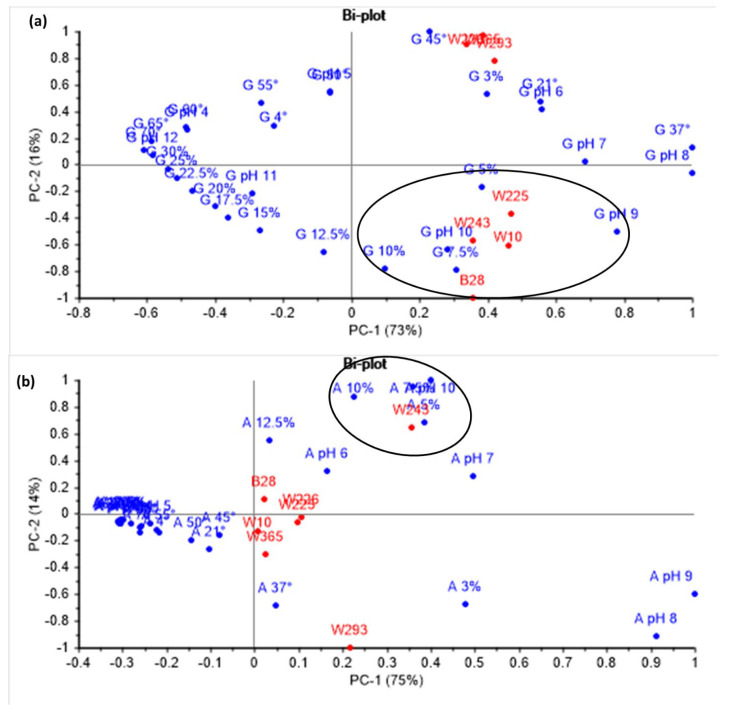
Principal Component Analysis (PCA) of growth (**a**) and amylase production (**b**) by seven isolates from Kuwait Sabkha. The letter “G” denotes growth and “A” denotes amylase production, both shown in blue. Growth or amylase production at different temperatures is indicated by G or A followed by 4 °C, 21 °C, 37 °C, 45 °C, 50 °C, 55 °C, 60 °C, 65 °C, and 70 °C. Growth or amylase production at different salt concentrations is represented by G or A followed by 3%, 5%, 7.5%, 10%, 12.5%, 15%, 17.5%, 20%, 22.5%, 25%, 27.5%, and 30%. Growth or amylase production at different pH levels is indicated by G or A followed by the pH range 4 to 12. The bacterial isolates are shown in red and include B28, W10, W225, W226, W243, W293, and W365. The circles in (**a**) highlight isolates that exhibit growth under extreme environmental conditions, while the circles in (**b**) indicate isolates that produce amylase under extreme conditions.

The dataset becomes complex with multiple variables (temperature, pH, and salt concentration) affecting the growth and amylase production of the seven isolates. In order to simplify this complexity and to select the best species for isolating a novel enzyme, a multivariate analysis was done using the growth and amylase production by the seven isolates at various temperatures, pH levels, and salt concentrations (Figure 2a,b).

Figure 2a shows the biplot of the principal component analysis (PCA) performed to explore how temperature, pH and salinity influence the growth pattern of these isolates. Principal Component 1 (PC1) explains 73% of the variance, while Principal Component 2 (PC2) accounts for 16%. Growth at varying pH levels and salt concentrations is primarily explained by PC1, while temperature-related growth is explained by PC2. Accordingly, the isolates cluster into two groups: one in the lower right quadrant and the other in the upper right quadrant. *Bacillus* sp. W225, *Priestia* sp. W243, *Marinobacter* sp. B28, and *Vibrio* sp. W10, which exhibits higher growth at pH 9–10, 5–10% salt concentrations, and 37 °C, are clustered in the lower right quadrant. In contrast, *Pseudomonas* sp. W226, *Halomonas* sp. W229, and *Bacillus* sp. W365, which show high growth at a 3% salt concentration, pH 6–7, and temperatures of 37–45 °C, are grouped in the upper right quadrant of the biplot. Many studies have isolated novel enzymes, such as halotolerant, acid-tolerant, alkaline-tolerant, and thermotolerant amylases, from *Bacillus*, *Marinobacter*, and *Pseudomonas* sp. [28,29,30,32].

A separate PCA was conducted to assess how the same environmental parameters affect the amylase production. Figure 2b displays the biplot of amylase production by the different isolates under various temperatures, pH, and salt concentrations, revealing a different pattern of influence compared to growth. PC1 and PC2 together explain 89% of the variance. The effect of temperature and pH on amylase production is explained by PC1, while PC2 explains the effect of salt concentration. Accordingly, most of the isolates are distributed near the center of the biplot, indicating low variability or deviation from the mean. *Priestia* sp. W243 and *Halomonas* sp. W293, which showed high amylase production, are positioned in the upper right quadrant and lower right quadrant along the positive PC1 axis of the biplot, respectively, indicating high variability. Of these two isolates, *Priestia* sp. W243 showed the highest amylase production at high salt concentrations (5–12.5%) and pH (7–10), while *Halomonas* sp. W293 showed high amylase production at a 3% salt concentration and a pH range of 8–9. Based on these observations and emerging research on *Priestia* sp. for its potential to produce novel enzymes [24,31,33], isolate W243 (*Priestia* sp.) has been selected as a candidate species for isolating novel enzymes that may be active at alkaline pH and high salt concentrations.

### 2.4. Isolation and Purification of α-Amylase with High Specific Activity from Priestia sp. W243

The purification steps for the novel high-specific-activity amylase are summarized in Table 2. Amylase from *Priestia* sp. W243 in the media supernatant (3 L) was concentrated to 30 mL by ultrafiltration. The ultrafiltration step resulted in a 9.25-fold purification due to the removal of lower molecular weight non-amylase proteins, peptides, and salts through a 5 kDa membrane.

**Figure 3:**
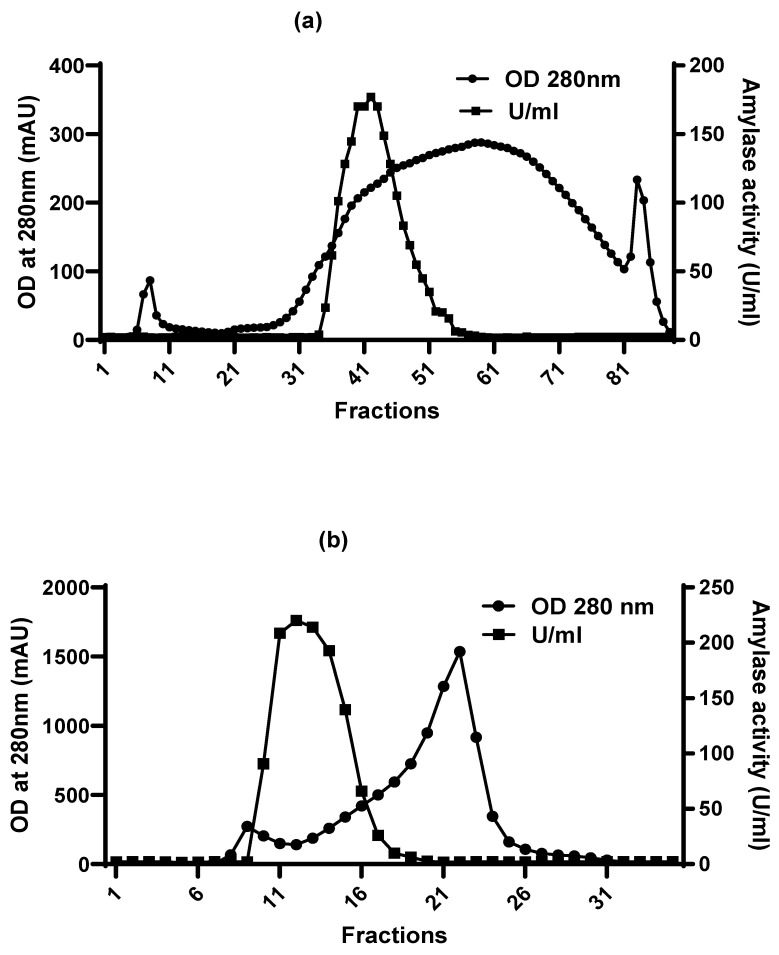
Purification profile of α-amylase from *Priestia* sp. using Foresight Nuvia Q anion exchange chromatography (**a**), followed by size exclusion chromatography with P-60 resin gel filtration column (**b**). Both columns were equilibrated with 25 mM Tris-HCl buffer at pH 9.1.

This process effectively increased the specific activity of amylase, as demonstrated by the reduction in total protein content, while retaining enzymatic activity in the concentrated retentate. Ultrafiltration primarily serves as a concentration step based on molecular weight cut-off and does not resolve proteins according to charge or size, resulting in a heterogeneous protein mixture. Hence, further purification was carried out using anion exchange chromatography (AEC) (Figure 3a), resolving into three peaks: two sharp and one broad. Amylase was eluted at the beginning of the broad peak, as confirmed by the amylase assay on the collected fractions (Figure 3a).

Anion exchange chromatography separates proteins based on their net negative charge at the working pH, enabling selective binding and elution of the target enzyme while removing charge-based contaminants. This step improved the enzyme’s specific activity to 6312.6 U/mg protein, resulting in a 15.6-fold purification with a yield of 21.29%. The amylase fraction from the AEC step was then subjected to size exclusion chromatography (Figure 3b). This further refines the purification by resolving proteins according to their molecular size, thereby producing a more homogeneous enzyme population. This step resolved two peaks, removing a significant impurity peak and yielding amylase with a higher specific activity. The final isolated enzyme exhibited a high specific activity of 8112.1 U/mg protein, achieving a 20-fold purification with a yield of 7.1% (Table 2).

**Figure 4:**
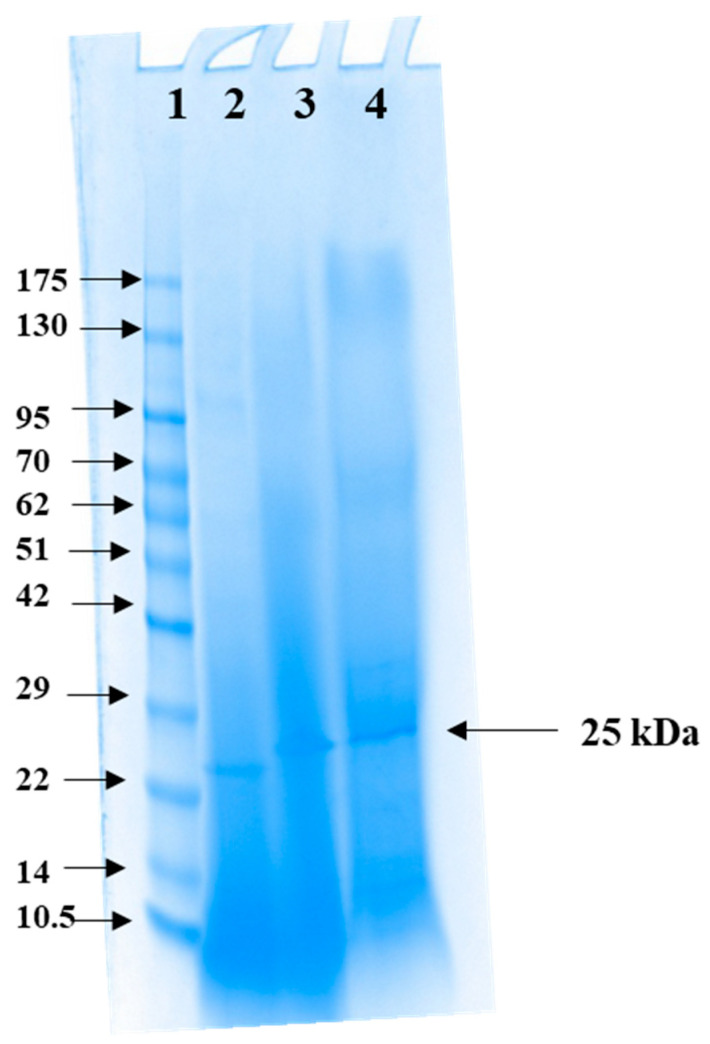
SDS-PAGE of the purified amylase from *Priestia* sp. W243. Lane 1—CSL-PPL Pre-stained protein ladder (Cleaver Scientific Ltd., Rugby, UK), Lane 2—Ultrafiltration Fraction, Lane 3—Anion Exchange Chromatography Fraction, Lane 4—Size Exclusion Chromatography Fraction.

α-amylase isolated from *Priestia* sp. (W243) has a molecular mass of 25 kDa, as determined by sodium dodecyl sulfate-polyacrylamide gel electrophoresis (SDS-PAGE) (Figure 4). This finding is consistent with a previous study by Ubi et al. [28], which reported a molecular mass of 25 kDa for α-amylase from *Priestia flexa* UCCM 00132. In contrast, α-amylases produced by various *Bacillus* species exhibit a wider range of molecular masses, ranging from 22 kDa to 149 kDa [29,34,35,36]. This consistency in molecular mass among *Priestia* spp. suggests a conserved structural feature specific to this genus.

### 2.5. Characterization of Purified α-Amylase

#### 2.5.1. Optimal Temperature and Thermal Stability

**Figure 5:**
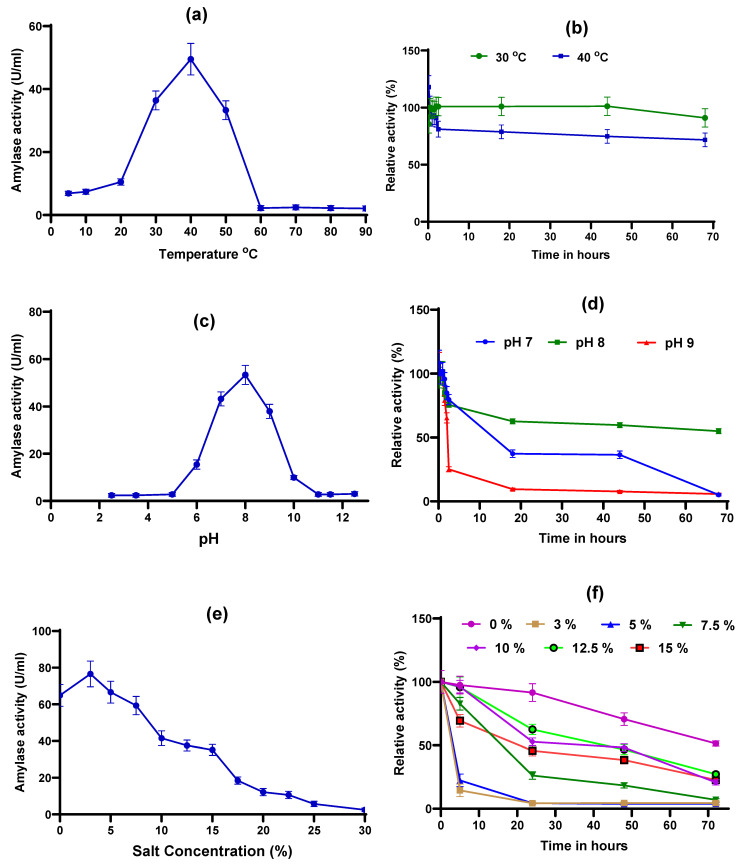
Optimum conditions and stability of purified α-amylase from *Priestia* sp. W243: (**a**) optimum temperature, (**b**) thermal stability around the optimum temperature, (**c**) optimum pH, (**d**) pH stability around the optimum pH, (**e**) optimum salt concentration, and (**f**) salt stability around the optimum salt concentration.

The purified enzyme exhibited high activity within a temperature range of 30 to 50 °C, with an optimal temperature of 40 °C (Figure 5a). The enzyme retained around 70% of its activity at 30 and 50 °C. However, activity decreased sharply at temperatures above 50 °C, with a 95% loss observed between 60 °C and 90 °C. At temperatures between 4 and 20 °C, the enzyme showed only 10 to 20% of its activity. In a study by Ubi et al. [24], the optimum temperature for a novel α-amylase from *Priestia flexa* UCCM 000132 was reported to be between 50 and 70 °C and the amylase retained 70% activity at 40 °C and 80 °C. Most amylases produced by *Bacillus* spp. are reported to have an optimal activity ranging from 40 to 70 °C [29,36,37].

The stability of the purified amylase was studied at 30 and 40 °C (Figure 5b). The amylase isolated from *Priestia* sp. W243 remained stable at 30 °C until 44 h, retaining over 97% of its activity and gradually declining thereafter. Even after 68 h, approximately 80% of the activity was preserved. However, at 40 °C, the enzyme remained stable for 2 h with over 90% activity, declining to 70% at 68 h. α-amylase isolated from *Priestia flexa* UCCM 000132 was reported to be stable between 55 and 65 °C for 2.5 h, with 70% activity remaining after 3.5 h [24]. However, amylase from *B. atrophaeus* NRC1 was reported to be stable below 50 °C, with significant activity loss at higher temperatures, where 35 and 67% loss in enzyme activity was recorded after enzyme incubation for 30 min at 60 °C and 70 °C, respectively [29]. Other studies on amylase from different *Bacillus* sp. also reported similar or lower stability for amylase [33,37]. Thus, the optimum temperature range of 30–40 °C and higher thermal stability at 30–40 °C of the purified α-amylase from *Priestia* sp. W243 makes this enzyme well-suited for industries involving moderate-temperature processes, such as baking and brewing. So the higher temperatures in the subsequent processes used in these industries can effectively inactivate the enzymes.

#### 2.5.2. Optimal pH and pH Stability

The enzyme showed high activity in the pH range of 7 to 9, with an optimal pH of 8 (Figure 5c). A sharp decline in activity was observed outside this pH range. In contrast, a novel tripartite amylase isolated from *Priestia flexa* UCCM 000132 was reported to be active over a wide pH range from 4 to 11 [24]. The optimal pH for amylases isolated from different *Bacillus* spp. has been reported to range from pH 6 to 10 [29,33,36,37].

pH stability of the purified α-amylase was investigated at three different pH levels viz. 7, 8, and 9 for 68 h (Figure 5d). The enzyme showed a high level of stability for about one hour across all tested pH levels, with notable stability at pH 8. The enzyme retained more than 85% activity for 2.5 h at pH 7 and 8, and 1.5 h at a pH of 9, indicating that it can perform effectively in neutral to mildly alkaline environments but with a shorter duration of stability as pH increases. At pH 8, the enzyme retained over 55% of its activity even after 68 h. The activity declined sharply after 1.5 h and 2 h at a pH of 9 and 7, respectively, and by the end of the 68 h period, the enzyme retained only 5% of its activity at both pH 7 and 9. This significant reduction in stability indicates a threshold beyond which the enzyme’s structural integrity and catalytic efficiency are compromised. The stability of enzymes at different pH levels is influenced by factors such as ionization of amino acid side chains, changes in electrostatic interactions, and alterations in hydrogen bonding networks within the protein structure [38]. Understanding these molecular mechanisms is crucial for elucidating the pH-dependent stability of enzymes and optimizing their performance across diverse applications.

#### 2.5.3. Optimal Salt Concentration and Salt Stability

The purified α-amylase was assayed for activity at different percentages of salt (0–30%), to identify the optimal salt concentration for its activity. The enzyme showed high activity under a wide range of salt concentrations from 0 to 15% with an optimal salt concentration of 3% (Figure 5e). This shows that the purified α-amylase is moderately halophilic. Even in the presence of 15% salt, the amylase retained more than 45% activity. Thereafter, the activity declined sharply at higher salt concentrations. Amylases from halotolerant *Bacilli* have been reported to show activity in salt concentrations ranging from 6% to 9% [18], suggesting that the α-amylase from *Priestia* sp. W243 has a broader functional range, showing potential applications in the processing of salted foods to enhance texture and flavor. It also shows promise for use in processing saline waters or managing waste solutions rich in starch residues and high salt content, providing practical solutions for industries operating in saline or high-salt conditions.

The stability of the purified amylase under varying salt concentrations (0 to 15%) was studied (Figure 5f). The enzyme was stable without any added salt for 24 h, retaining more than 90% activity and around 50% activity even after 72 h (Figure 5f). Although the enzyme activity was highest in the presence of 3% salt, this amylase was not stable under 3% and 5% salt concentrations. A significant loss of activity (*p* < 0.0001) was observed after 5 h, with 85.5% and 77.6% loss at 3% and 5% salt concentration, respectively. Almost 95% of the activity was lost by 24 h under these salt concentrations. Interestingly, better stability was observed at salt concentrations of 7.5% to 15%. The highest stability was observed at 10% and 12.5% salt concentrations, where there was no significant loss (*p* > 0.05) of activity within five hours. In addition, this enzyme retained 53% and 63% activity at 10 and 12.5% salt concentrations, respectively, even after 24 h.

Halophilic enzymes display distinctive structural and catalytic characteristics that enable activity under conditions of high salinity and low water availability. Although the molecular principles governing halophilic protein adaptation are not yet fully resolved, several studies have identified common strategies that support enzyme stability and function in saline environments [39]. These include an enrichment of negatively charged residues on the protein surface, reduced surface hydrophobicity, and an increased prevalence of salt bridges. Together, these features promote protein solvation and structural integrity at high ionic strength. However, such adaptations are not universal among halophilic proteins [40,41], underscoring the structural diversity within this group and the need for protein-specific analyses.

In the present study, the purified α-amylase exhibited notable stability even in the absence of salt. A similar observation was reported for α-amylase isolated from the halophilic archaeon *Haloarcula hispanica* [42]. In that study, the enzyme contained a moderately elevated proportion of acidic amino acid residues (16.2%) relative to non-halophilic counterparts, though this proportion was lower than that reported for α-amylase from the haloalkaliphilic archaeon *Natronococcus amylolyticus* (24.3%). The authors suggested that this intermediate level of acidic residues may allow retention of enzymatic structure and activity in the absence of salt [42]. In contrast, a high density of acidic residues in many halophilic proteins can lead to destabilization at low salt concentrations due to increased electrostatic repulsion [43].

A defining feature of halophilic proteins is the reduced abundance of bulky hydrophobic residues on their surface, accompanied by a relative enrichment of smaller and borderline hydrophobic amino acids [43,44,45,46]. This compositional bias contributes to increased molecular flexibility, enhanced surface hydration, and diminished hydrophobic interactions, all of which are favorable under high-salt conditions. Additionally, the formation of salt bridges between oppositely charged residues plays a central role in stabilizing protein folding, maintaining oligomeric assemblies, and preserving catalytic competence. Consistent with these structural features, many halophilic enzymes lose stability when salt concentrations fall below 1–2 M [45]. This behavior may explain the reduced stability of the α-amylase from this study at lower salinity (3–5%) and its enhanced stability at higher salt concentrations (10–15%). Structural studies of other halophilic enzymes provide further support for this interpretation. For example, malate dehydrogenase from *Haloarcula marismortui* contains an increased number of salt bridges relative to non-halophilic homologs, contributing to enhanced stability at high salinity; the enzyme exists as a tetramer under these conditions but dissociates into monomers as salt concentration decreases [47,48]. Similarly, isocitrate dehydrogenase from *Haloferax volcanii* is active as a dimer at high salt levels but undergoes irreversible inactivation at low salinity due to dissociation into partially folded monomers [49]. The spectroscopic investigations of ferredoxin from *Halobacterium salinarum* revealed that increasing salt concentrations reduce electrostatic repulsion through ion binding, thereby stabilizing the oligomeric structure required for catalytic activity [50]. Overall, these findings emphasize the critical role of salt-mediated electrostatic interactions and oligomeric stabilization in halophilic enzymes. Hence, a detailed structural study of the α-amylase from *Priestia* sp. W243 is necessary to understand its biochemical and biophysical properties.

#### 2.5.4. Effect of Metal Cations and Metal Chelators on the Purified α-Amylase

**Figure 6:**
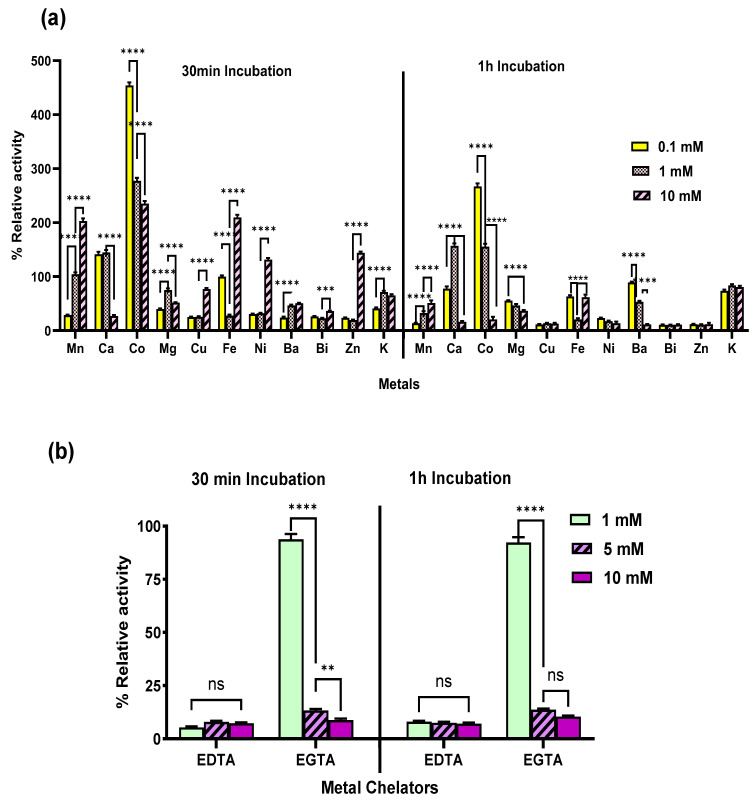
Effect of (**a**) metals (**b**) metal chelators on the activity of purified α-amylase from *Priestia* sp. W243. Where, **** *p* < 0.0001, *** *p* < 0.0007, ** *p* < 0.005, ns—non significant.

Studies on the effect of metal cations on purified enzymes are very important for understanding the regulation and optimization of enzyme activity in biological systems and industrial applications [51]. The results of different metal cations on purified amylase revealed stimulatory and inhibitory effects on the enzyme activities (Figure 6a). Though dose-dependent, Mn^2+^, Ca^2+^, Co^2+^, Fe^2+^, Ni^2+^, and Zn^2+^ had a stimulatory effect on the enzyme. Of these, Mn^2+^, Fe^2+^, Ni^2+^, and Zn^2+^ exhibited inhibitory effects at lower concentrations but stimulated enzyme activity at higher concentrations.

Incubation at 10 mM for 30 min with these metals enhanced activity by 202.9%, 209.6%, 131.3%, and 143.8%, respectively (Figure 6a). Ca^2+^ and Co^2+^ ions enhanced activity at lower concentrations, and their stimulatory effects decreased at higher concentrations. Interestingly, Co^2+^ at 0.1 mM exhibited a remarkable stimulatory effect, increasing activity by 454.2% (Figure 6a). A dose-dependent trend was observed in extended 60 min incubation (Figure 6a). This finding underscores the potential of Co^2+^ as a potent activator of amylase activity. This dose-dependent response in amylase activity with different metals suggests a complex interaction between the metal ions and the enzyme, potentially involving changes in the enzyme’s conformation or active site accessibility [51]. A similar observation of the stimulatory effect of Co^2+^ has been reported for Bacillus amyloliquefaciens [52]. The association of divalent Co^2+^ ions with the surface of the α-amylase macromolecule was reported to promote higher catalytic activity by enhancing structural flexibility, as indicated by reduced Tm values [52].

Previous studies reported that Ca^2+^ activates amylases from various *Bacillus* species [18,53]. Most amylases are metalloenzymes that require Ca^2+^ ions for activity, stability, and structural integrity [53]. The stimulation of enzyme activity by Ca^2+^ has been attributed to the formation of a calcium-sodium-calcium metal triad at the enzyme’s main Ca^2+^ binding site, bridging its A and B domains [54]. However, the α-amylase in the present study was activated more by Co^2+^ than by Ca^2+^, suggesting that this enzyme may act in a Co^2+^-dependent manner. To find this, the effect of different metal chelators, such as EDTA and EGTA, on the activity of the purified enzyme was studied (Figure 6b). A drastic inhibition of amylase was observed with EDTA, and there was no significant difference (*p* > 0.05) among the different concentrations tested. EDTA is a strong metal chelating agent that binds various divalent cations, including Ca^2+^, Mg^2+^, Mn^2+^, Zn^2+^, Co^2+^, and others [55]. Interestingly, EGTA retained 94% activity at 1 mM concentration, but a significant decrease in activity (*p* < 0.0001) was observed as the concentration increased (Figure 6b). EGTA was reported to be more selective for Ca^2+^ over other divalent cations [56]. The significant decrease in activity at higher concentrations of EGTA suggests that at higher concentrations, it was chelating other divalent cations also [57,58]. The specific inhibition of purified amylase by EDTA at 1 mM concentration but not by EGTA suggests that the enzyme’s activity depends on cations other than Ca^2+^, such as Mn^2+^, Co^2+^, Fe^2+^, Ni^2+^, or Zn^2+^, which are effectively chelated by EDTA but not as effectively by EGTA. Since Ca^2+^ and Co^2+^ are the two metals that have stimulatory effects on the purified enzyme (Figure 6a), it implies that the enzyme likely requires these divalent cations for its structural stability or catalytic activity. This, along with the lack of enzyme inhibition upon removal of Ca^2+^ by EGTA, suggests that the enzyme may be cobalt-dependent.

Metals such as Mg^2+^, Ba^2+^, Bi^2+^, and K^+^ showed inhibitory effects in both 30- and 60 min incubations, with the extent of inhibition varying in a concentration-dependent manner (Figure 6a). At 10 mM concentration, the inhibitory effects for these metals were 51.7%, 50.6%, 36.1%, and 65.1%, respectively. Contrary to the results of the present study, a study on amylase from *Bacillus atrophaeus* NRC1 reported that Ca^2+^, Mg^2+^, and Ba^2+^ ions stimulated activity by 141.6%, 128.7%, and 113.5%, respectively. While Cu^2+^, K^+^, Ni^2+^, and Zn^2+^ inhibited activity with inhibitions of 51.8%, 15.8%, 31.9%, and 54.7%, respectively [29]. Interestingly, Co^2+^ and Mn^2+^, which stimulated α-amylase activity in the present study, were reported to inhibit amylase from *Bacillus* sp. altogether [29]. In the present study, Cu^2+^ inhibited enzyme activity at all three concentrations tested (Figure 6a). Vieille and Zeikus [59] reported that Cu^2+^ ions can promote the autooxidation of cysteines, resulting in the formation of intra- and intermolecular disulfide bridges or sulfenic acid. Similarly, the inhibition of enzymatic activity by Cu^2+^ observed in this study, as was also reported for amylase from *Bacillus atrophaeus* NRC1, indicates the possible involvement of sulfhydryl groups in the enzyme’s catalytic site [29]. Thus, the stimulatory and inhibitory effects of metal cations on the purified amylase provide valuable insights into optimizing metal ion concentrations to enhance enzyme activity or to regulate it through inhibitory metals. Such understanding can enable precise control in industrial and biotechnological applications, particularly in the food and beverage industries. Additionally, the significant stimulation observed with Co^2+^ at 0.1 mM highlights its potential as an activator, making it promising for applications like bioremediation or processes requiring enhanced enzymatic efficiency in the presence of specific metal ions.

#### 2.5.5. Effect of Detergents on the Activity of Purified α-Amylase

**Figure 7:**
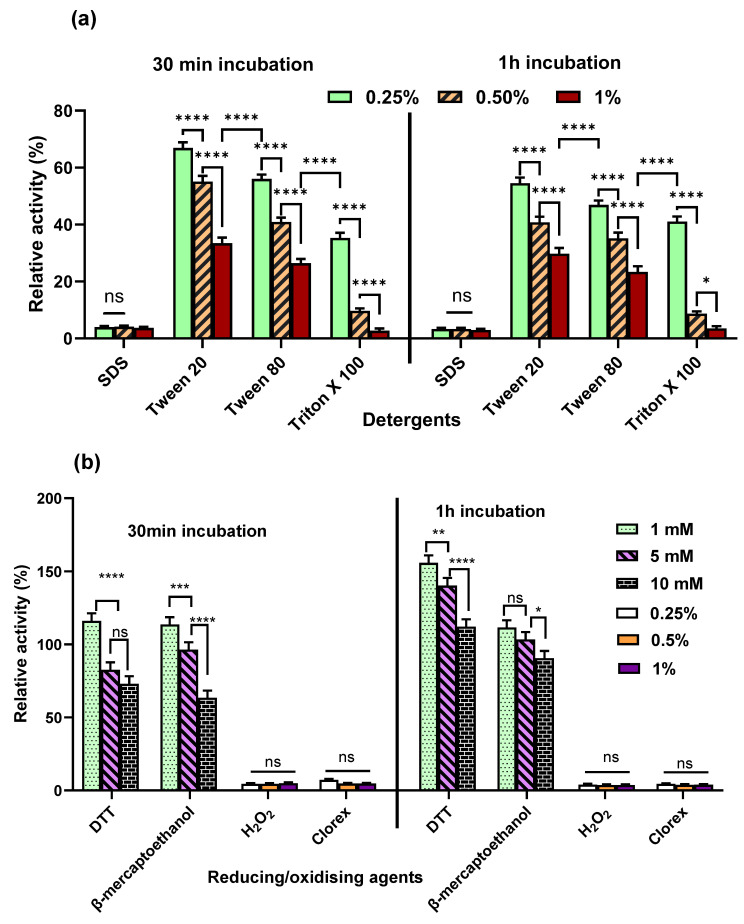
Effect of (**a**) Detergents, (**b**) oxidizing and reducing agents on the activity of purified α-amylase from *Priestia* sp. W243. Where, **** *p* < 0.0001, *** *p* = 0.0003, ** *p* = 0.001, * *p* = 0.037, ns—non significant.

Among the detergents tested, the anionic surfactant SDS significantly (*p* < 0.0001) inhibited purified α-amylase, reducing its activity to only 3–4% at all concentrations and incubation periods tested (Figure 7a). This observation supports the previous findings where SDS nearly completely suppressed α-amylase activity from *Bacillus thermoleovorans* [60]. The ionic surfactant, such as SDS, can induce local conformational alterations in the enzyme’s active site even at very low concentrations, leading to partial unfolding and inactivation [61].

In contrast, nonionic detergents such as Tween 20, Tween 80, and Triton X-100 retained 67%, 56%, and 30% enzyme activity, respectively, at the lowest concentration tested. However, increasing concentrations caused a significant (*p* < 0.0001) decline in activity for all the detergents, with Triton X-100 showing the most pronounced effect, reducing activity to 2% at the highest concentration and prolonged incubation periods (Figure 7a). Unlike SDS, nonionic detergents lack charge and, at lower concentrations, do not disrupt ionic or electrostatic interactions, which are crucial for maintaining the enzyme’s structural integrity and active site conformation [62]. However, at higher concentrations, structural alterations caused by these detergents become severe, leading to enzyme inactivation. The stronger inhibition by Triton X-100 at higher concentrations than Tween 20 and Tween 80 (Figure 7a) is likely attributed to its distinct physicochemical properties. Triton X-100 contains a hydrophobic aromatic group (octylphenyl moiety) attached to a polyoxyethylene chain, whereas Tween 20 and Tween 80 are composed of polyoxyethylene sorbitan with long fatty acid chains (lauric acid and oleic acid, respectively) [63]. Triton X-100’s stronger hydrophobic interactions and a higher tendency for micelle formation may destabilize the enzyme or block access to the active site more effectively [64]. In contrast, Tweens exert a gentler effect on protein stability due to weaker hydrophobic interactions and less pronounced micelle formation [65].

#### 2.5.6. Effect of Reducing Agents and Oxidising Agents on the Activity of α-Amylase

The oxidizing agents such as Clorox and H_2_O_2_ inhibited the enzyme drastically and retained only 3–7% activity at different concentrations, regardless of the incubation time (Figure 7b). This is likely due to the oxidation of methionine or cysteine residues near the catalytic site. It is well established that hydrogen peroxide and other oxidizing agents can oxidize methionine or cysteine residues located in the active sites of many enzymes, and such oxidative modification often leads to a reduction or complete loss of enzymatic activity [66,67]. Yang et al. [68] reported that the oxidative stability and catalytic efficiency of alkaline α-amylase from *Alkalimonas amylolytica* were significantly enhanced under H_2_O_2_ stress by protein engineering, in which five oxidation-prone methionine residues in the active site were substituted with oxidation-resistant amino acids such as threonine, isoleucine, and alanine.

In contrast, the activity of purified amylase increased in the presence of reducing reagents, DTT, and β-mercaptoethanol. The stimulation was higher when incubated for 60 min for both agents (Figure 7b). A similar protective effect of reducing agents has been reported for α-amylase isolated from Bacillus licheniformis CUMC305 [69] and for extracellular endoglucanase from Myceliophthora thermophila D-14 [70]. This enhancement may be attributed to the prevention of methionine oxidation by reducing agents such as DTT and β-mercaptoethanol, as oxidation of methionine residues in the active site has been shown to cause loss of α-amylase functionality [68].

Many enzymes contain disulfide bonds (–S–S–) between cysteine residues that are essential for maintaining their native three-dimensional structure. Reducing agents such as β-mercaptoethanol and DTT cleave these disulfide bonds to generate free thiol groups, which can stabilize enzymes that require reduced cysteine residues for activity. Conversely, reduction in disulfide bonds may inactivate enzymes whose structural integrity or catalytic function depends on intact disulfide linkages [71]. Therefore, the observed activation of this α-amylase suggests that its disulfide bonds are not critical for maintaining the global fold of the enzyme. Instead, these disulfide bonds may be surface-exposed or serve a regulatory role by restricting conformational flexibility near the active site. Their reduction may relieve structural constraints, increase active-site accessibility, and thereby enhance catalytic efficiency. In addition, reducing agents help maintain cysteine residues in their reduced thiol state, preventing oxidative inactivation and preserving residues essential for catalysis [72,73]. The higher stimulation observed with prolonged incubation suggests that reducing disulfide bonds may require more time to take full effect [57,74]. The greater activation observed with DTT compared to β-mercaptoethanol during prolonged incubation may be attributed to the stronger reducing capacity of DTT [75,76]. Similar enhancement of enzyme activity upon reduction has been reported for enzymes containing redox-sensitive cysteine residues, indicating that disulfide bond reduction does not necessarily cause denaturation but can instead promote functional activation, depending on the structural context [71,76]. The enhanced activity in the presence of reducing agents in this α-amylase may find application in protein engineering techniques targeting disulfide bond formation or reduction.

## 3. Materials and Methods

### 3.1. Bacterial Isolates from Kuwait Sabkha

**Figure 8:**
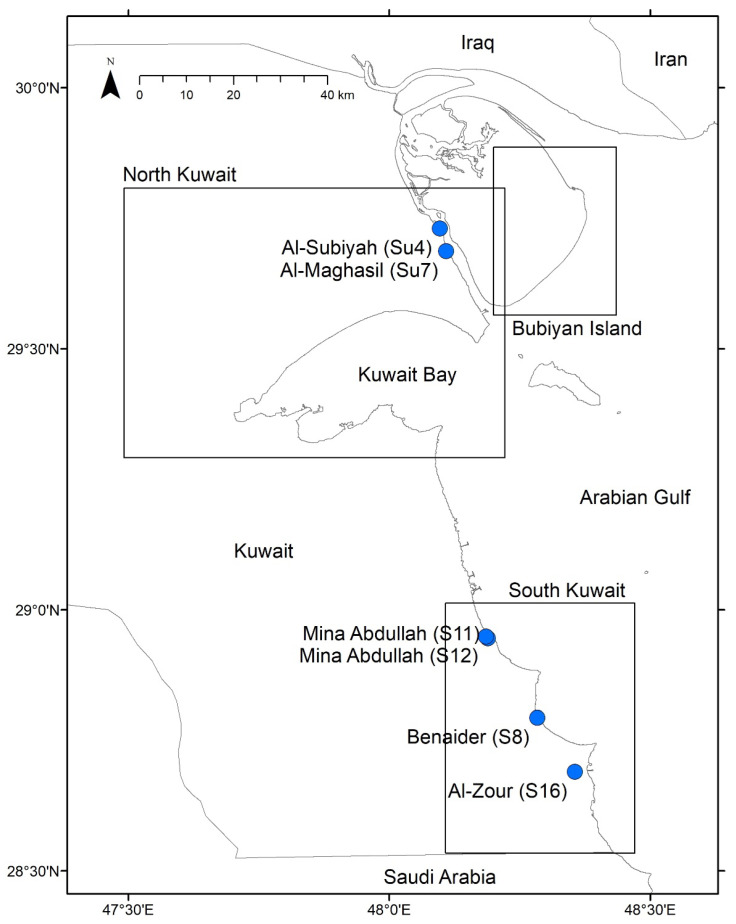
Geographical locations of the selected extreme coastal sabkha stations in Kuwait.

Seawater and sediment samples were collected from extreme coastal sabkha stations in Kuwait, namely Benaider, Mina Abdullah, Al-Zour, Al Subiya, Al-Maghasil, and Bubiyan Islands (Figure 8). All the chemicals were from Sigma-Aldrich unless specified otherwise (Steinheim, Germany). Halophilic bacteria from the samples were isolated, as described here. Samples (1 g or 1 mL) were serially diluted in minimal medium (Glucose 1%; KH_2_PO_4_ 0.1%; FeCl_2_.6H_2_O 0.001%, NH_4_Cl, 0.2%) supplemented with 3 and 15% (*w*/*v*) sea salt (Sigma S9883), and adjusted to three pH values: 5.5, 7, and 10. As the samples were collected between November 2021 and February 2022, during which the temperatures ranged from 15 to 26 °C, the initial enrichment was accordingly performed at 25 °C for 10 days. Appropriate dilutions (10^−1^ to 10^−4^) were inoculated in triplicates by spread plating in Marine Agar 2216 (MA, Difco, Birmingham, UK) plates (Peptone 5 g/L, Yeast extract 1 g/L, Ferric Citrate 0.1 g/L, Sodium Chloride 19.45 g/L, Magnesium Chloride 8.8 g/L, Sodium Sulfate 3.24 g/L, Calcium Chloride 1.8 g/L, Potassium Chloride 0.55 g/L, Sodium Bicarbonate 0.16 g/L, Potassium Bromide 0.08 g/L, Strontium Chloride 34 mg/L, Boric Acid 22 mg/L, Sodium Silicate 4 mg/L, Sodium Fluoride 2.4 mg/L, Ammonium Nitrate 1.6 mg/L, Disodium Phosphate 8 mg/L, Agar 15 g/L), supplemented with 3 and 15% sea salt, and adjusted to pH values of 5.5, 7, and 10 [77,78]. The plates were incubated at 25 °C for 10 days. Isolates with unique colony morphology (round, round with wavy margins, wavy, slimy, dry) and pigmentation (white, off-white, light pink) representing the diverse group of bacteria from this environment were purified and stored. The isolates were initially screened for extracellular enzyme activities, including protease, lipase, amylase, and DNase [9]. Seven isolates that grew under extreme pH, salt, or temperature conditions and exhibited high amylase activity were selected for the present study. Enzyme activity was classified based on the clearance zone size as low (≤5 mm), medium (6–10 mm), high (11–15 mm), and very high (>15 mm). The selected isolates showed clearance zones greater than 16 mm and were obtained from Bnaider, Mina Abdullah, Al-Zour, and Bubiyan Island.

### 3.2. Identification of Amylase-Producing Isolates

Identification of amylase producers was carried out by the 16S rRNA sequence method. The genomic DNA of these cultures was isolated by GenElute bacterial genomic DNA kit (Sigma-Aldrich, St. Louis, MO, USA) as per the manufacturer’s instructions. DNA was amplified for their 16S rRNA gene, followed by sequence analysis. Briefly, the full-length 16S rRNA region of the bacterial isolates was amplified using the primer 27F (5′-AGAGTTTGATCCTGGCTCAG-3′) and 1492R (5′-GGTTACCTTGTTACGACTT-3′) [79]. Polymerase Chain Reaction (PCR) reaction was carried out in a 30-µL reaction mixture containing 15-µL master mix (GoTaq, Promega, Madison, WI, USA) and 1 µL each forward and reverse primer (10 µM) (Macrogen, Seoul, Republic of Korea), and 1 µL template (50 ng/μL). The PCR (GeneAmp PCR system 9700, Applied Biosystem, Foster City, CA, USA) conditions used consisted of 2 min denaturation at 94 °C, 45 s at 94 °C, 1 min at 59 °C, and 2 min 30 s at 72 °C for 30 cycles. This was followed by extension for 10 min at 72 °C [9]. The amplified DNA fragments were analyzed through 1% agarose gel electrophoresis and documented using a Bio-Rad gel documentation system (Bio-Rad, Hercules, CA, USA). The PCR products were sent to a third-party sequencing partner in India. The PCR products were purified using a BioRad spin column and sequenced at BGI, Hong Kong. The service provider carried out post-bioinformatics analysis. The 16S rRNA sequence was submitted to the Nucleotide Basic Local Alignment Search Tool (BLAST), and complete sequence alignments were performed using PHYDIT. Phylogenetic analysis was performed with PHYLIP, using the neighbor-joining method [9,80,81,82]. The identified halophile sequences were deposited in the National Center for Biological Information (NCBI) GenBank database (SUB4973142).

### 3.3. Optimal Temperature, pH, and NaCl Concentrations on α-Amylase Production by the Seven Selected Isolates

To determine the optimum temperature of amylase production, all seven selected isolates were inoculated in 5 mL marine broth (MB, Oxoid, Basingstoke, UK), and incubated at different temperatures (4, 22, 37, 45, 50, 55, 60, 65, and 70 °C) for 48 h. The optimum pH for the enzyme production by the selected isolates was studied by adjusting the pH of the MB to different levels (pH 3 to 12.5) using appropriate buffers. The buffers employed were 25 mM citrate buffer (pH 3, 4, 5 and 6), 25 mM phosphate buffer (pH 7), 25 mM Tris-HCl buffer (pH 8 and 9), 25 mM Glycine NaOH buffer (pH 10), phosphate NaOH buffer (pH 11), 25 mM KCl-NaOH buffer (pH 12, 12.5 and 13). The temperature and pH study used marine broth supplemented with 3% NaCl for all cultures except for B28. Isolate B28 was studied by inoculating it in marine broth with 15% NaCl. The optimum NaCl for the enzyme production was studied by supplementing different concentrations of NaCl (*w*/*v*) to the marine broth medium (3, 5, 7.5, 10, 12.5, 15, 17.5, 20, 22.5, 25, and 30%). Luria-Bertani (LB) broth was used to study amylase production without salt. All the pH and salt study tubes were incubated at 37 °C for 48 h. The initial and final OD at 600 nm was measured to determine the growth during the above experiment. After incubation, 1 mL of the culture was transferred to a 1.5 mL Eppendorf tube and centrifuged at 10,000 rpm for 1 min. The cell-free supernatant was subjected to amylase activity under standard assay conditions. The activity was expressed as a percentage relative to amylase activity [83].

### 3.4. Amylase Activity Assay

The starch hydrolytic activity of the culture supernatant/enzyme was estimated using the 3,5-dinitro salicylic acid (DNS) assay method [83] with some modifications to the assay in a microplate reader. For this, 75 µL of enzyme solution was mixed with 75 µL of 1% starch solution as substrate and incubated at 40 °C for 30 min. The released sugar was measured by adding 0.15 mL of 1% DNS in 0.4 M NaOH, followed by boiling for 5 min to develop color. Then, 42 µL of 40% potassium sodium tartrate was added. The absorbance of the mixture (200 µL) was measured at 540 nm in a multimode microplate reader (Varioscan™ Lux, Thermo Fisher Scientific, Vantaa, Finland). One unit of amylase activity is defined as the amount of enzyme that releases one micromole of reducing sugar (as glucose equivalents) per minute under specific assay conditions.

### 3.5. Culturing the Most Potent α-Amylase Producer for Enzyme Isolation

A loopful of 18 h culture of the most potent amylase producer (*Priestia* sp. W243) was inoculated into 30 tubes of 5 mL marine broth supplemented with 7.5% NaCl (*w*/*v*) adjusted to a pH of 9 to develop the inoculum. Marine broth without added starch was used to assess basal extracellular amylase production, in order to avoid substrate interference during enzyme activity assays. These tubes were then incubated at 37 °C for 24 h with shaking at 110 rpm. This was used as the seed culture to inoculate into thirty 500 mL bottles containing 100 mL of the above medium and incubated at 110 rpm at 37 °C for 4 days. The cell-free supernatant containing amylase was harvested by centrifugation at 3500 rpm for 10 min at 4 °C and subjected to isolation, partial purification, and characterization of amylase.

### 3.6. Purification of α-Amylase from Priestia sp. W243

The crude enzyme from the culture supernatant was subjected to three-step purification viz. ultrafiltration, anion exchange, and size exclusion chromatography as described below. The cell-free culture supernatant was subjected to ultrafiltration using a solvent-resistant stirred cell (MilliporeSigma, Søborg, Denmark) with 76 mm 5 kDa cellulose ultrafiltration discs (Amicon, Bedford, MA, USA). A nitrogen pressure of 0.4 MPa was applied to the stirred cell and the permeate was discarded.

The concentrated crude enzyme was then subjected to anion-exchange chromatography by fast protein liquid chromatography (FPLC) (NGC Discover™ 100, Bio-Rad, Hercules, CA, USA). The column used was Foresight™ Nuvia™ Q (5 mL, Bio-Rad, Herules, CA, USA). It was initially equilibrated with 25 mL of 25 mM Tris-HCl buffer, at a pH of 9.1 (Buffer A), with a flow rate of 5 mL/min. The sample was adjusted to pH 11.5 to 12 to ensure the effective binding to the anion exchange column (at lower pH values, the sample did not bind efficiently and was lost during the washing step). One mL of the sample was injected into the column and washed with 15 mL of Buffer A and eluted with 60 mL of Buffer B (25 mM Tris HCl containing 1 M NaCl, pH 9.1) in a gradient ranging from 0 to 50%. Following the gradient elution, an isocratic elution was performed with 15 mL of 100% Buffer B. Subsequently, the column was washed with 15 mL of Buffer A. One milliliter fraction was collected by a fraction collection unit (Bio-Rad, Hercules, CA, USA). All the fractions collected were assayed for amylase activity. As mentioned above, fractions containing *α*-amylase activity were pooled together and concentrated by ultrafiltration.

The concentrated enzyme was subjected to size exclusion chromatography by fast protein liquid chromatography (FPLC) (NGC Discover 100, Bio-Rad, Hercules, CA, USA). The column used was a Bio-scale column, MT20 (20 mL, Bio-Rad, Hercules, CA, USA), with P-60 resin. The column was equilibrated with 10 mL of 25 mM Tris-HCl buffer pH 9.1, with a flow rate of 0.3 mL/min. The sample in one ml quantity was injected and eluted by using the same buffer at a flow rate of 0.3 mL/min using 60 mL of the buffer. One milliliter fraction was collected using a fraction collection unit (Bio-Rad, Hercules, CA, USA). As described before, fractions containing *α*-amylase activity were pooled together and concentrated by ultrafiltration. The purified enzymes were used for further characterization studies.

### 3.7. Protein Content and SDS PAGE Electrophoresis

The protein content of the cell-free extract and enzyme at different stages of purification was determined by the Lowry method [84] with bovine serum albumin as a standard. The molecular mass of the isolated amylase was determined by running a sodium dodecyl sulfate–polyacrylamide gel electrophoresis (SDS-PAGE), employing a Mini-PROTEAN^®^ TGX™ (Tris-Glycine eXtended, Bio-Rad, Hercules, CA, USA) precast gels, any kDa. Ten microlitres of the samples mixed with non-reducing loading buffer were loaded onto the gel. Electrophoresis was performed using a Bio-Rad Mini Protean-Tetra Cell with a voltage of 200 V for 45 min and Tris Glycine SDS (TGS) buffer (Bio-Rad 161-0732, Hercules, CA, USA) was used as the running buffer. CSL-PPL pre-stained protein ladder, ranging from 10 to 175 kDa, was used as markers. The gel was stained overnight in a solution of Coomassie Brillant Blue R-250 (0.24%) and destained for 5 h in destaining solution (1:1:4 methanol:glacial acetic acid:water). The protein bands were visualized using Bio-Rad Gel Doc system.

### 3.8. Characterization of Purified α-Amylase

The purified novel amylase from *Priestia* sp. W243 was characterized to identify optimum temperature, pH, and salt and was studied for its stability and the effect of metals and inhibitors on its activity. The amylase enzyme was diluted to 350 fold and used for characterization assays.

#### 3.8.1. Effect of Temperature, pH, and Salt Concentration on the Activity and Stability of the Purified α-Amylase

The optimum temperature of the purified enzyme was determined by measuring its activity after incubation with a 1% starch substrate at various temperatures (4, 10, 22, 30, 40, 50, 60, 70, 80, and 90 °C) for 30 min. A 25 mM phosphate buffer (pH 7) was used during this experiment. The thermal stability of the isolated enzyme was studied by preincubating it within its optimal temperature range (30 °C and 40 °C) over varying durations (30 min to 68 h), followed by measuring the residual activity under standard assay conditions.

The optimum pH of the purified enzyme was determined by incubating the enzyme with 1% starch (30 min) at the optimum temperature of 40 °C in respective buffers corresponding to the pH range from 3 to 12.5 and measuring the activity under standard assay conditions. Buffers used included 25 mM citrate buffer (pH 3, 4, 5, and 6), 25 mM phosphate buffer (pH 7), 25 mM Tris-HCl buffer (pH 8 and 9), 25 mM glycine-NaOH buffer (pH 10), phosphate-NaOH buffer (pH 11), and 25 mM KCl-NaOH buffer (pH 12, 12.5). The pH stability was assessed by pre-incubating the purified enzyme at pH values 7, 8, and 9 for 30 min to 68 h, followed by measuring the residual activity under standard assay conditions.

The optimum salt concentration for the purified enzyme was assessed by incubating it (30 min) with 1% starch at 40 °C at various salt concentrations (0–30%) and measuring its activity under standard assay conditions. Salt stability was assessed by pre-incubating the purified enzyme at 0, 3, 5, 7.5, 10, 12.5, and 15% for 30 min to 68 h and measuring the residual activity under standard assay conditions.

#### 3.8.2. Effect of Metal Ions on the Activity of Purified α-Amylase

The influence of exogenously added metal ions on the activity and stability of the purified enzyme was studied at metal concentrations of 0.1, 1, and 10 mM. The metal ions tested included Mn^2+^, Ca^2+^, Co^2+^, Mg^2+^, Cu^2+^, Fe^2+^, Ni^2+^, Ba^2+^, Bi^2+^, Zn^2+^, and K^+^ (MnSO_4_·H_2_O, CaCl_2_·2H_2_O, CoCl_2_·6H_2_O, MgCl_2_·6H_2_O, CuSO_4_, FeSO_4_·7H_2_O, NiSO_4_·7H_2_O, BaCl_2_·2H_2_O, BiN_3_O_4_·5H_2_O, ZnSO_4_·7H_2_O, KCl). The purified enzyme in Tris-HCl buffer (pH 8.0) was pre-incubated at 40 °C with these metals for 30 and 60 min. Enzyme activity in the absence of metal ions served as a control. For each metal ion and concentration tested, parallel negative controls containing the corresponding salts but without enzyme were included to account for any non-specific reduction in the DNS reagent. Residual enzyme activity was then measured under standard assay conditions. Results are expressed as a percentage relative activity.

#### 3.8.3. Effect of Detergents, Oxidizing Agents, Chelators, and Reducing Agents on the Activity of Purified α-Amylase

The effect of various detergents, oxidizing agents, chelators, and reducing agents on the activity of purified enzyme was studied. Detergents studied include Tween 20, Tween 80, sodium dodecyl sulfate (SDS), and Triton X-100 at 0.25%, 0.5%, and 1%. Oxidizing agents such as hydrogen peroxide (H_2_O_2_) and Clorox (sodium hypochlorite) were studied at concentrations of 0.25%, 0.5%, and 1%. The chelators studied include ethylenediaminetetraacetic acid (EDTA) and ethylene glycol-bis (β-aminoethyl ether)-N,N,N′,N′-tetraacetic acid (EGTA) at a concentration of 1, 5, and 10 mM. Reducing agents, such as dithiothreitol (DTT) and β-mercaptoethanol, were tested at 1, 5, and 10 mM concentrations. The purified amylase was pre-incubated individually in Tris-HCl buffer (pH 9.0) with these compounds for 30 and 60 min. Residual enzyme activity was then measured under standard assay conditions. The enzyme activity in the absence of these agents served as a control. Results are expressed as a percentage relative activity.

### 3.9. Data Analysis

Results were presented as mean ± SD. The data on the growth and amylase production by different isolates, as well as the stability and characterization studies of the enzyme, were subjected to two analyses of variance (ANOVA) using the statistical package program GraphPad Prism 10 (Graphpad Software Inc., San Diego, CA, USA; Version 10.6.1). The statistical comparisons among the groups were done using Tukey’s multiple comparison test, and a *p*-value < 0.05 was considered statistically significant. The principal component analysis (PCA) was also performed to evaluate the relationship between different amylase-producing isolates and their growth and amylase production under varying environmental conditions. Full cross-validation was applied, and all variables were standardized by weighting them as 1/standard deviation. The PCA analysis was conducted using Unscrambler X software (CAMO Software, Oslo, Norway; Version 10.5).

## 4. Conclusions

In conclusion, our evaluation of seven bacterial species isolated from Kuwait’s sabkhas under varying temperatures, pH, and salinity conditions identified *Priestia* sp. W243 as the most promising candidate for amylase production, particularly under alkaline and high-salt conditions. The isolation and purification of amylase from *Priestia* sp. W243 yielded an enzyme with high specific activity (8112.1 U/mg protein), optimal activity at 40 °C and pH 8, and stability across a broad range of salt concentrations. The activity of this enzyme was enhanced by several cations, including Mn^2+^, Ca^2+^, Co^2+^, Fe^2+^, Ni^2+^, and Zn^2+^, while being inhibited by Cu^2+^, Mg^2+^, Ba^2+^, Bi^2+^, and K^+^. Co^2+^, followed by Ca^2+^, exhibited a strong stimulatory effect on the purified α-amylase. The specific inhibition by EDTA, but not by EGTA, along with the pronounced stimulatory effect of Co^2+^, suggests that this α-amylase may be a cobalt-dependent metalloenzyme, emphasizing its potential applications in bioremediation. Inhibition of the enzyme by detergents and oxidizing agents, along with the stimulatory effect of reducing agents, indicates that disulfide bonds and thiol groups are essential for preserving its structural integrity and catalytic function. Overall, *Priestia* sp. W243 from Kuwait’s sabkhas emerges as a robust and versatile source of a novel amylase with significant biotechnological potential, particularly for industrial processes requiring stability and efficiency in extreme conditions.

## Figures and Tables

**Table 1:** Identification of Amylase Producer from Kuwait’s Sabkha by 16S rRNA Gene Sequence Analysis and Their Close Match Type Strains (% Similarity).

Code of Isolate	Source	Accession No. ^a^	Nearest NCBI Match ^b^	Accession No. ^c^	% Similarity ^d^
W10	Kuwait: Benaider	OQ777182	*Vibrio nereis*	NR_118092.1	82.93
W225	Kuwait:Mina Abdulla	OQ777231	*Bacillus subtilis*	OM535929.1	89.87
W226	Kuwait:Mina Abdulla	OQ777232	*Pseudomonas* sp.	MF148487.1	90.78
W243	Kuwait:Mina Abdulla	OQ777251	*Priestia flexa*	MK012676.1	91.97
W293	Kuwait:Mina Abdulla	OQ777268	*Halomonas ventosae*	AB617544.1	89.72
W365	Kuwait:Al-Zour	OQ777306	*Bacillus velezensis*	MW494547.1	91.19
B28	Kuwait:Bubiyan	OQ777471	*Marinobacter guineae*	NR_042618.1	88.42

^a^ GenBank accession no. of our strains deposited on NCBI website (http://www.ncbi.nlm.nih.gov/pubmed accessed on 7 April 2023). ^b^ Closest species with highest % identity and highest Max score on BLAST search. ^c^ GenBank accession number of the closest relative on NCBI website. ^d^ Based on BLAST search results, identity (%) of strains compared to the closest relatives.

**Table 2:** Purification Scheme for Amylase from *Priestia* sp. W243 from Kuwait’s Sabkha.

Purification Steps	Total Activity (U ^a^)	Total Protein (mg)	Specific Activity (U/mg of Protein)	Recovery (%)	Purification Fold
Crude enzyme	101,687.4	250.8	405.5	100	1
Ultrafiltration	45,868.9	12.2	3750.5	45.10	9.25
Anion Exchange Chromatography	21,652.3	3.43	6312.6	21.29	15.6
Size Exclusion Chromatography	7219.8	0.89	8112.1	7.1	20

^a^ One unit of amylase activity was defined as the amount of enzyme that releases one micromole of reducing sugar (as glucose equivalent) per minute under specific assay conditions.

## Data Availability

All data supporting this study are included in the manuscript. Further inquiries can be directed to the corresponding authors.
